# Penoscrotal Extension and Fistulation From Urothelial Carcinoma of the Bladder

**DOI:** 10.7759/cureus.73206

**Published:** 2024-11-07

**Authors:** Anna Akpala, Suzanne Dunk, Debashis Sarkar

**Affiliations:** 1 Urology, Queen Elizabeth Hospital Birmingham, Birmingham, GBR

**Keywords:** bladder mass, muscle invasive bladder cancer, transurethral resection of bladder tumor, urethrocutaneous fistula, urothelial bladder cancer, vesico-cutaneous fistula

## Abstract

The commonest malignancy of the urinary tract is bladder cancer, with the commonest presentation being painless visible haematuria. Just like other malignancies, it can spread, commonly to surrounding tissues like the prostate, seminal vesicles, and vagina, distantly to lymph nodes, lungs, liver, and bone, and less commonly to the skin and subcutaneous tissues. This is a case of a man with muscle-invasive bladder cancer who underwent radical radiotherapy. He presented nine days into the course of his radiotherapy with new symptoms of pain, swelling, and discharge, particularly at the penoscrotal junction.

Ultrasound scans of the testes were normal, and he was treated as a case of scrotal skin abscess with antibiotics. His symptoms persisted and worsened over time despite treatment and multiple hospital attendances. He developed a discharge of fluid from multiple sinuses in the area. Further cross-sectional imaging and direct visualisation with cystoscopy led to the conclusion that there was a urethral recurrence of his urothelial carcinoma which had extended locally into the soft tissues in the penoscrotal area and caused urocutaneous fistulous tracts.

An extensive literature review showed no documented cases of vesicocutaneous fistula from urothelial carcinoma, making this the first reported case of penoscrotal extension of bladder cancer and fistulation after radiotherapy.

## Introduction

Bladder cancer is the most common malignancy of the urinary tract and the tenth most diagnosed cancer worldwide [1]. Tobacco smoking is the strongest risk factor for bladder cancer, which accounts for 50 - 60% of all cases [1]. Other risk factors include environmental and occupational exposure to carcinogens, such as aromatic amines, polycyclic aromatic hydrocarbons, hereditary and genetic factors, male gender, and obesity, among others [1].

About 20 - 25% of bladder cancers are muscle invasive at diagnosis [2] and 10 -15% are estimated to present with metastases [3]. The most common sites of metastases are the lymph nodes, liver, lungs, bone, and adrenals [4].

Around 2% of patients with urothelial malignancy of the urinary bladder will have a synchronous upper tract tumour and 6% will develop a metachronous upper tract lesion [5]. The rate of urethral recurrence was reported as 1.4 - 6.2% following radical cystectomy in one study [6]. The recurrence of urothelial carcinoma in the urethra following radical cystectomy has been reported as up to 10% in one study [7]. Another study published by Cresswell et al demonstrated a 4.7% rate of urethral recurrence following radical radiotherapy for bladder cancer after a 5-year follow-up period [8].

Scrotal skin abscesses are relatively common and usually uncomplicated. They are usually treated by incision and drainage, but if already discharging, this is not always necessary.

## Case presentation

**Figure 1:**
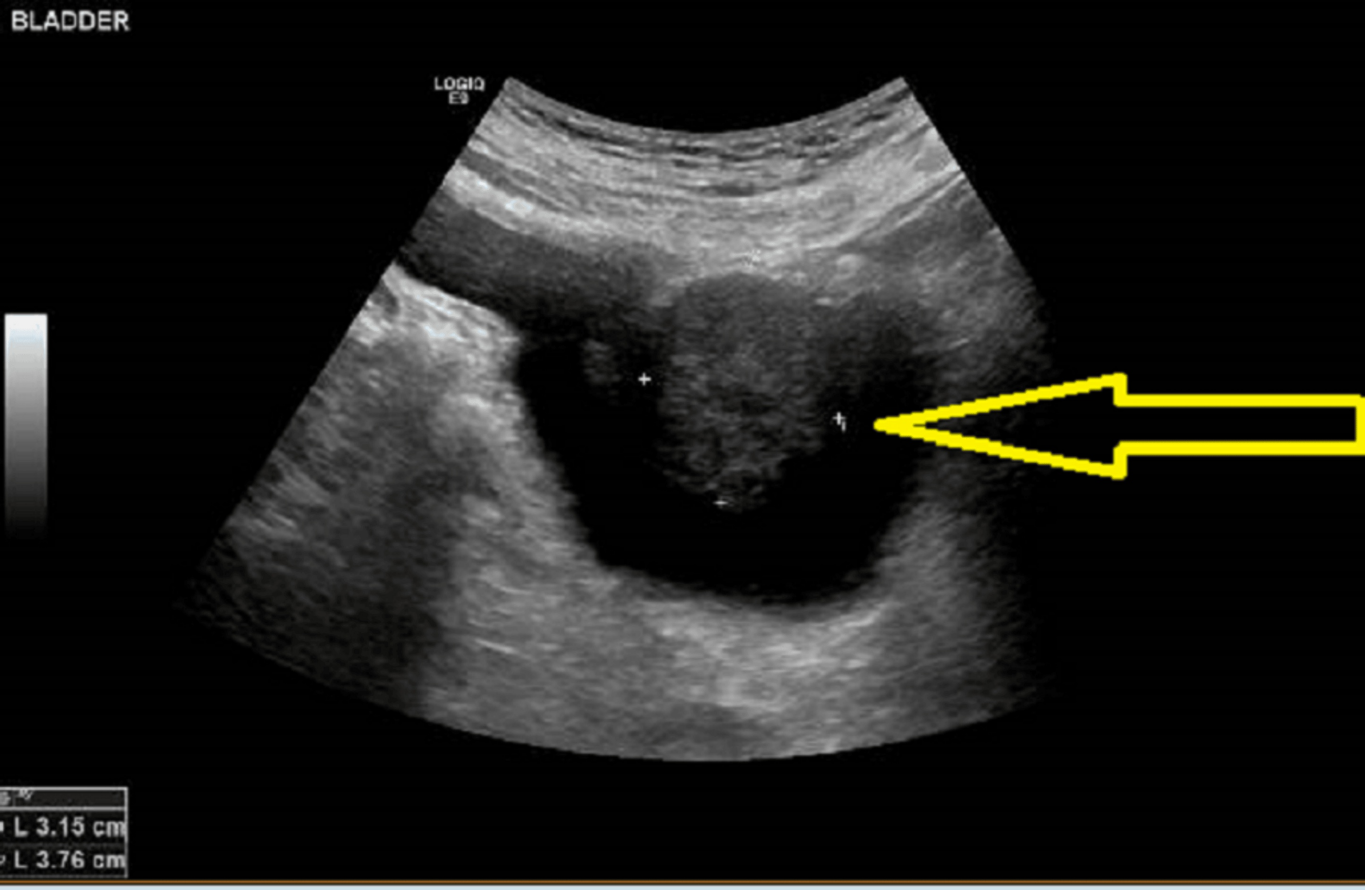
Ultrasound scan of the bladder showing a hypoechoic lesion in the bladder

**Figure 2:**
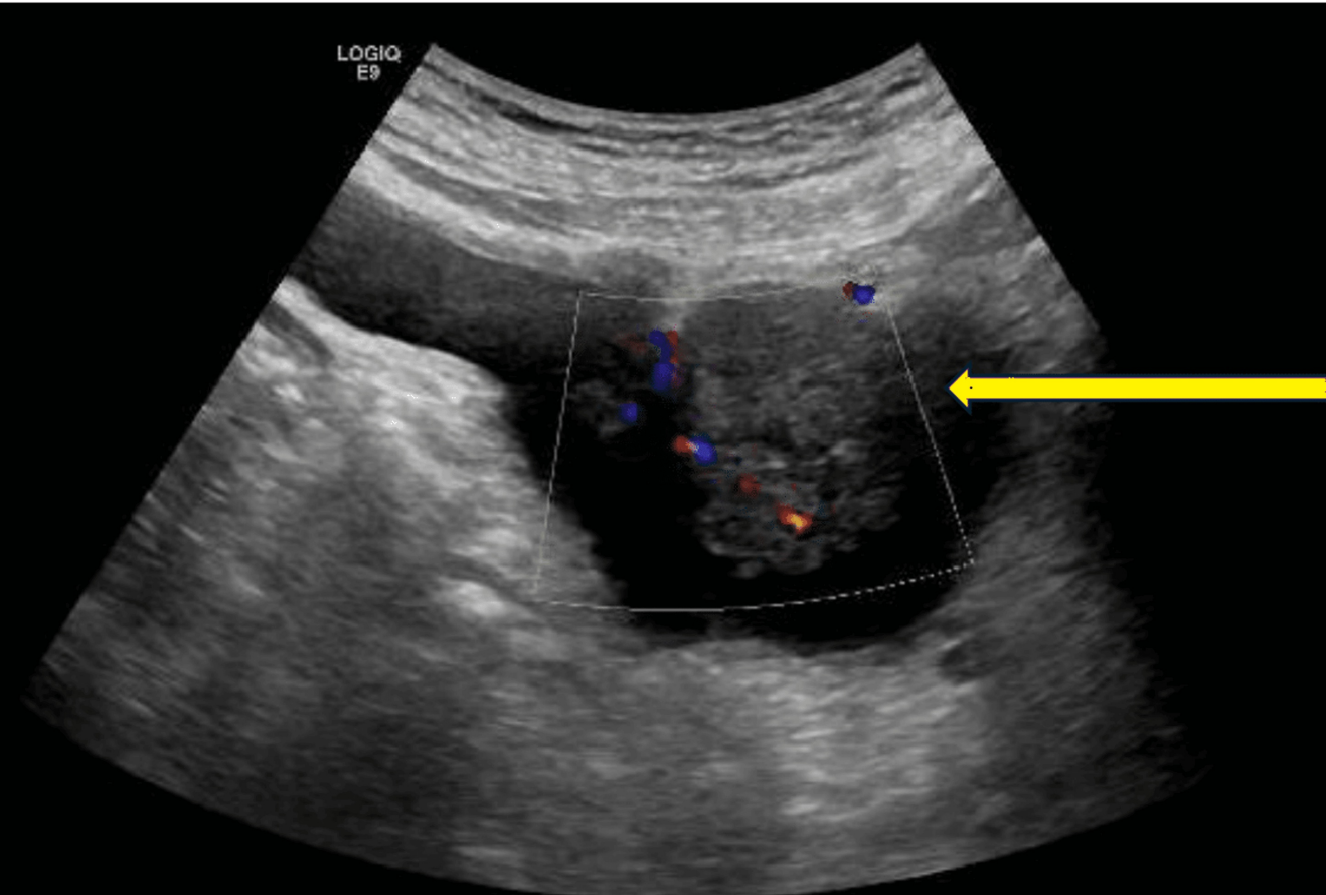
Ultrasound scan with Doppler views showing vascularity to the lesion

A man who was 71 years of age at the time, was first referred to our centre on a two-week wait pathway with visible haematuria and abdominal ultrasound scan findings of a 32 mm x 37 mm well-defined hypoechoic lesion in the bladder as shown in Figure 1 and Figure 2.

**Figure 3:**
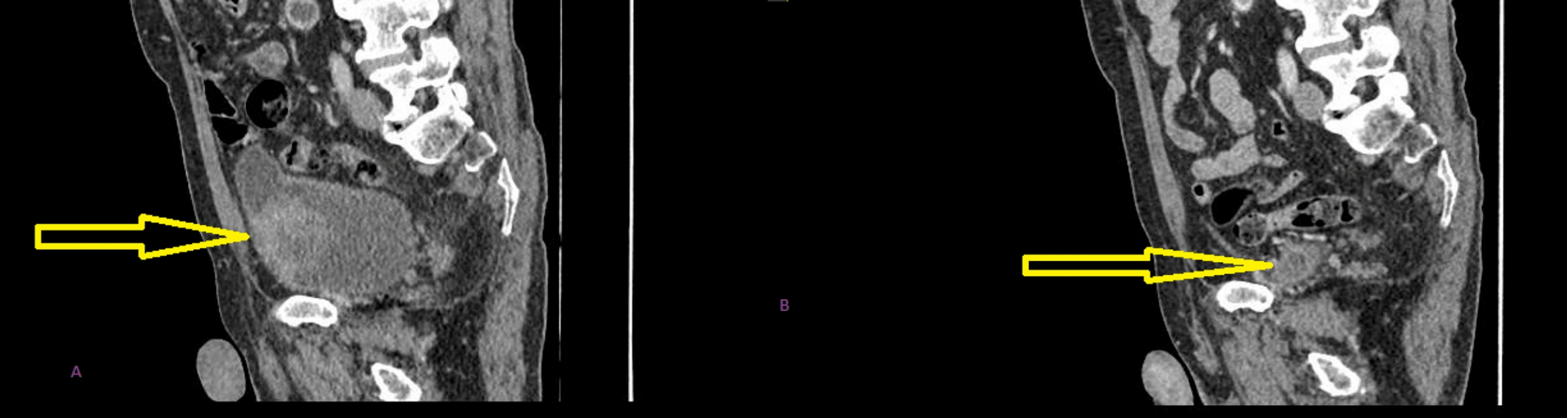
Sagittal view of CT scan before TURBT in figure A and after TURBT but prior to radiotherapy with urethral catheter in situ in figure B. The bladder mass is seen clearly in figure A, whereas in figure B, the mass can no longer be seen following TURBT. TURBT - transurethral resection of bladder tumor

**Figure 4:**
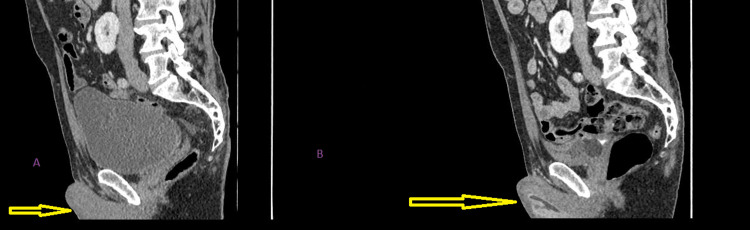
Cross sectional sagittal image before TURBT in figure A, and after TURBT but prior to radiotherapy with urethral catheter in situ in figure B. Penoscrotal area was disease free at the time as evidenced by maintenance of subcutaneous fat plane in both figures. TURBT- transurethral resection of bladder tumor

**Figure 5:**
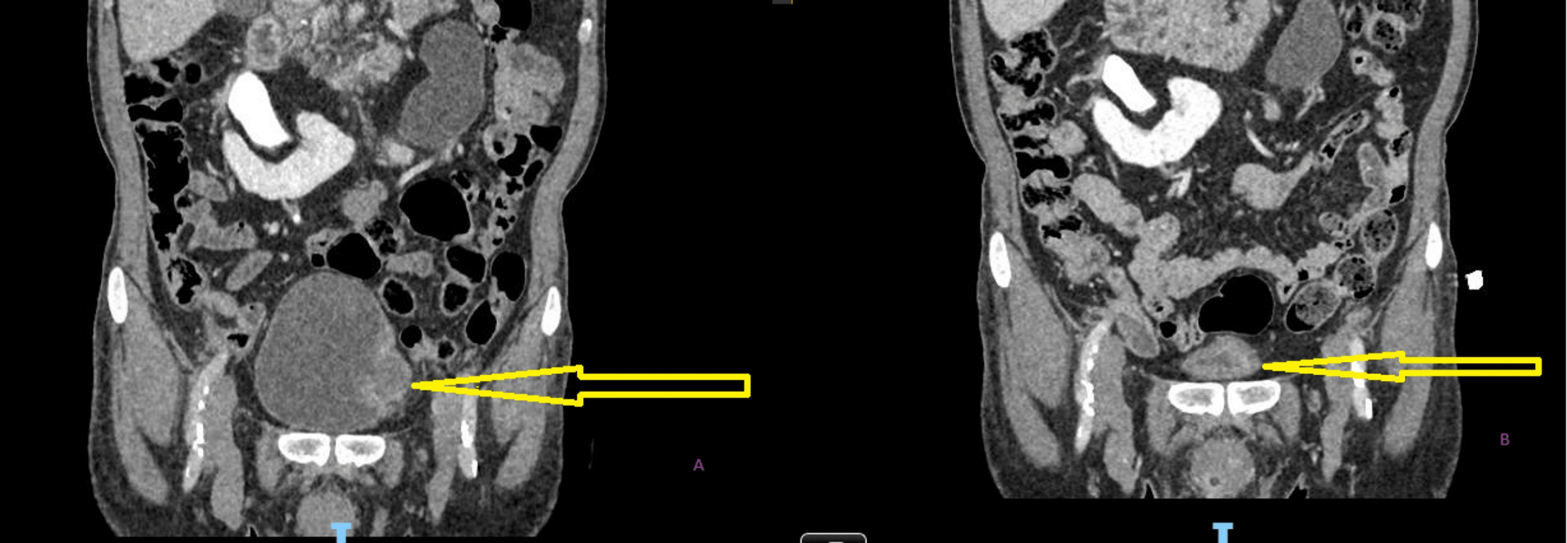
Cross-sectional coronal image of the penoscrotal area before TURBT in figure A, and after TURBT but prior to radiotherapy with urethral catheter in situ in figure B. The penoscrotal area were grossly normal in both figures. TURBT - transurethral resection of bladder tumor

**Figure 6:**
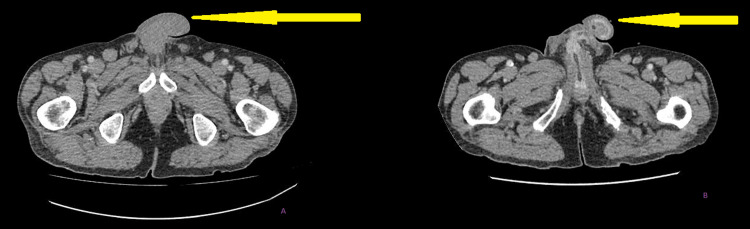
Cross-sectional axial image before TURBT in figure A and after TURBT but prior to radiotherapy in figure B. Penoscrotal area was disease free at the time as evidenced by maintenance of subcutaneous fat plane in both images. TURBT - transurethral resection of bladder tumor

His only risk factor for bladder cancer was being an ex-smoker. Flexible cystoscopy revealed a normal urethra, a moderately enlarged prostate, and a large bladder tumour of around 6 cm on the left lateral wall. Computed tomography (CT) scan of his urinary tract, thorax, abdomen, and pelvis at this time revealed a right-sided horseshoe kidney, no ureteric lesions, the bladder mass as well as one enlarged external iliac lymph node but no other lymphadenopathy or distant metastases (Figures 3-6, sagittal view, coronal view and axial views of his CT scan before and after transurethral resection of bladder tumour (TURBT)).

He underwent rigid cystoscopy and transurethral resection of bladder tumour (TURBT) which revealed a normal urethra, a 6 cm solid-looking bladder mass extending from the left lateral wall to the anterior wall with no other tumours seen. He had an uncomplicated TURBT and there were no bladder perforations. The histology revealed a high-grade solid urothelial carcinoma with squamous differentiation invading the detrusor muscle (G3 pT2 at least). Following multi-disciplinary team meetings and further discussions, he was deemed unfit for radical cystectomy due to extensive cardiac comorbidities and referred for radical radiotherapy. He was assessed by the oncologists who considered him to be too high risk for neoadjuvant or concurrent chemotherapy.

He had a repeat staging CT scan prior to the commencement of radiotherapy and this showed the same enlarged external iliac lymph node as before which appeared to have slightly decreased in size from 15mm to 13.9mm with no other lymphadenopathy or distant metastases.

Two weeks prior to the commencement of his radiotherapy, he had urethral catheterization done due to incidental finding of poor bladder emptying with a plan for trial without catheter (TWOC) on completion of treatment. He had no penoscrotal symptoms at this time and no abnormalities of the penoscrotal area were noted during catheterisation. He began treatment with 20 Fractions of 55 Gy radical radiotherapy over a four-week period. He had five fractions per week at 2.75 Gy per dose, using CT localisation on an empty bladder and utilising the volumetric modulated arc therapy (VMAT) technique. By this time, he was two months post-TURBT.

Nine days into the course of his radiotherapy, he complained of new symptoms of a discharging sinus around the scrotum. On examination, he also had a yellow discharge from his penile tip, with slough around the base of the penis with an approximately 3 cm oval mobile mass, minimally tender and surrounding erythema. Ultrasound scans of his testes were essentially normal, although the skin and soft tissues were not commented on.

He was treated as a case of discharging scrotal skin abscess with antibiotics. He continued and successfully completed his radiotherapy treatment at the same dose and without a break with a total treatment time of four weeks as originally planned.

Six weeks after completion of his radiotherapy he again presented with persistent swelling, pain, and discharge at the penoscrotal junction. However, he had also developed a skin sinus that seemed to occasionally discharge what appeared to be urine. On examination, an indurated area was noted at the penoscrotal junction with discharging abscess, the testes were normal and uninvolved. He underwent another ultrasound scan which again showed normal testes with no comment on skin and soft tissues and was again managed as unresolving scrotal abscess.

**Figure 7:**
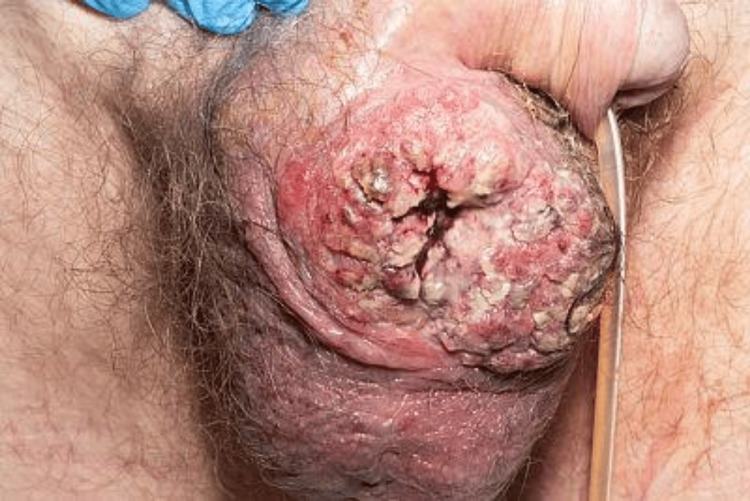
Fungating scrotal mass with urethral catheter in situ.

A further seven weeks later he presented again with worsening symptoms including discharge of urine from four places in the scrotum. On examination, he had a fungating scrotal mass which appeared to be discharging urine (Figure 7).

**Figure 8:**
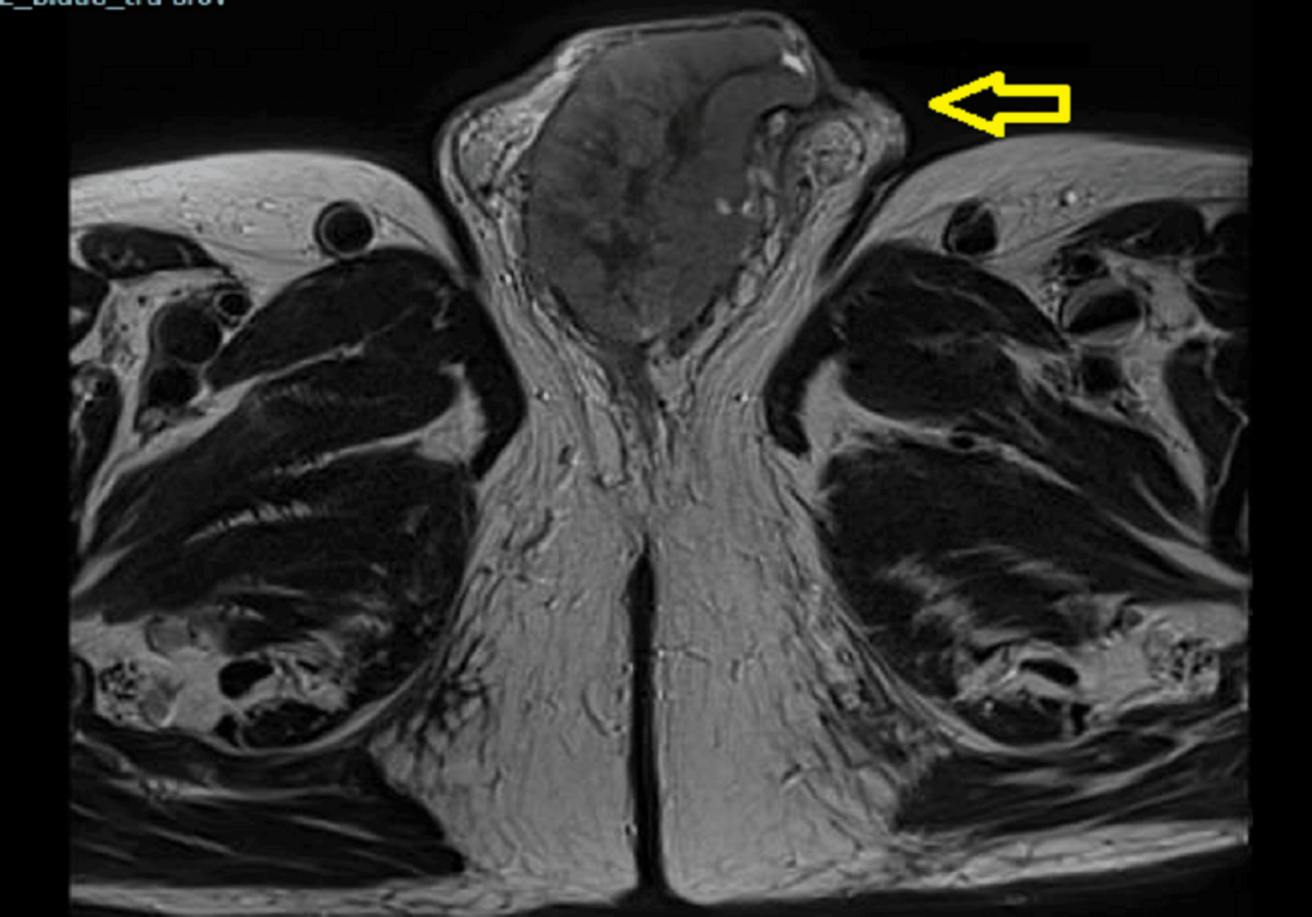
T2-weighted MRI of the pelvis showing a large penis (73.2 mm x 47.2 mm) poorly enhancing encapsulated mass within the inferior margin of the corpus spongiosum

**Figure 9:**
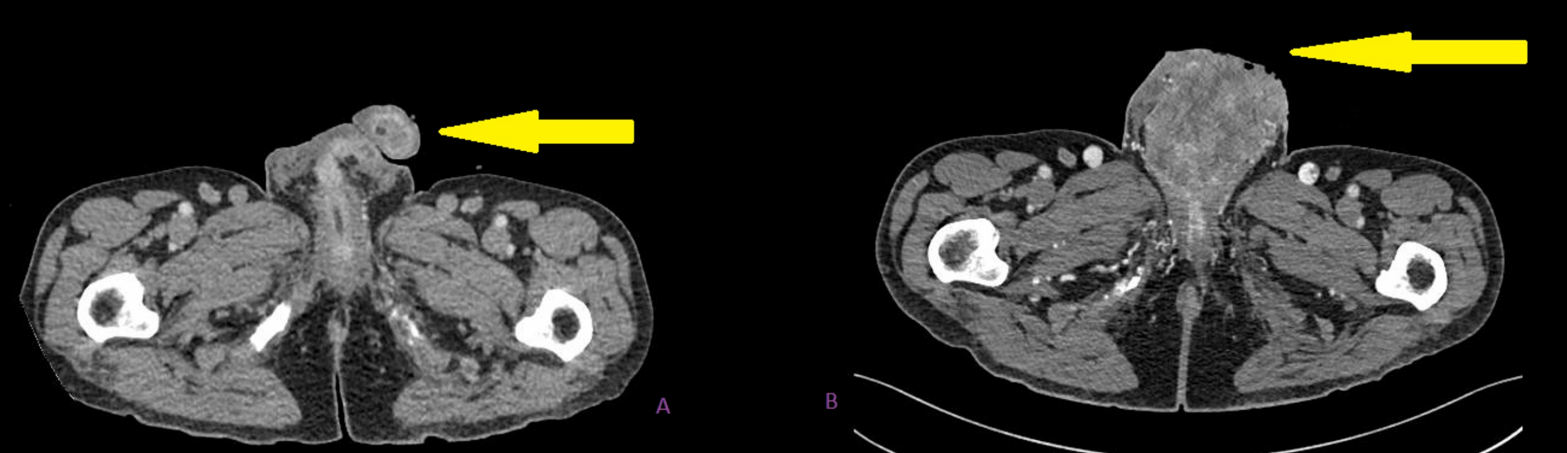
Cross-sectional axial image before radiotherapy in figure A and after radiotherapy in figure B showing the anatomy of the penoscrotal region after radiotherapy distorted by the huge mass.

**Figure 10:**
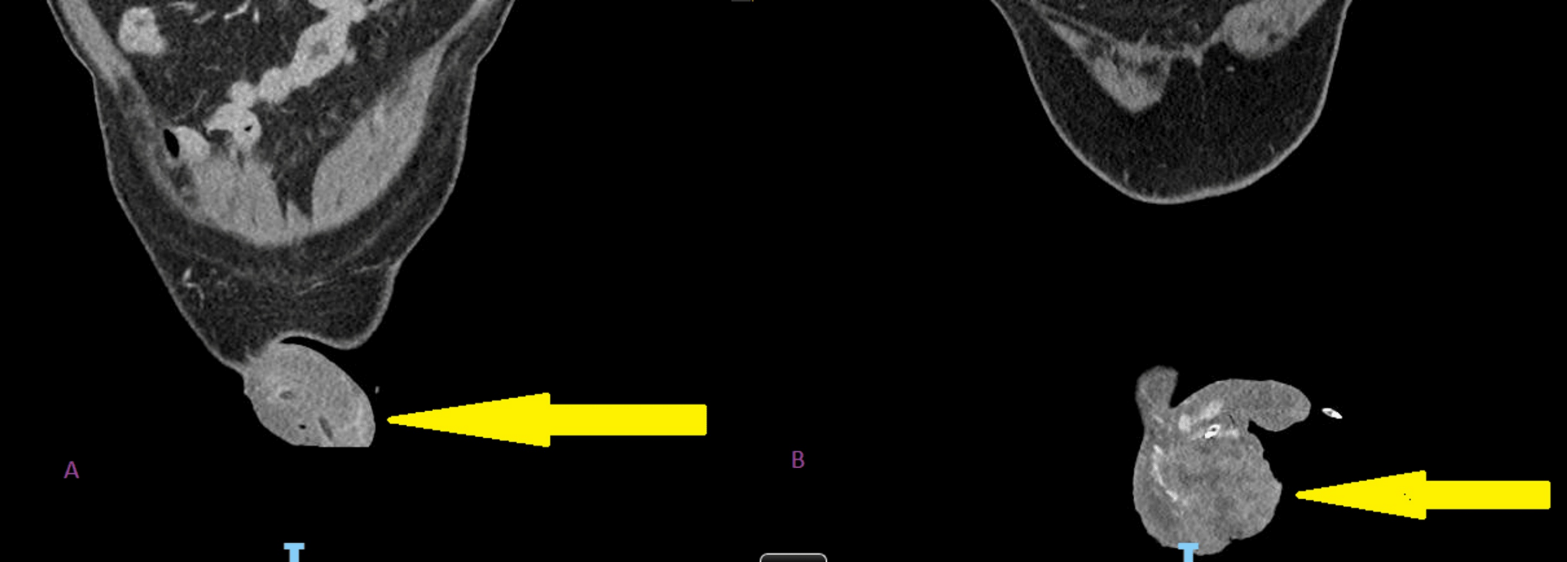
Cross-sectional coronal image before radiotherapy in figure A and after radiotherapy in figure B showing the anatomy of the penoscrotal region post radiotherapy distorted by the huge mass with a urethral catheter in situ.

**Figure 11:**
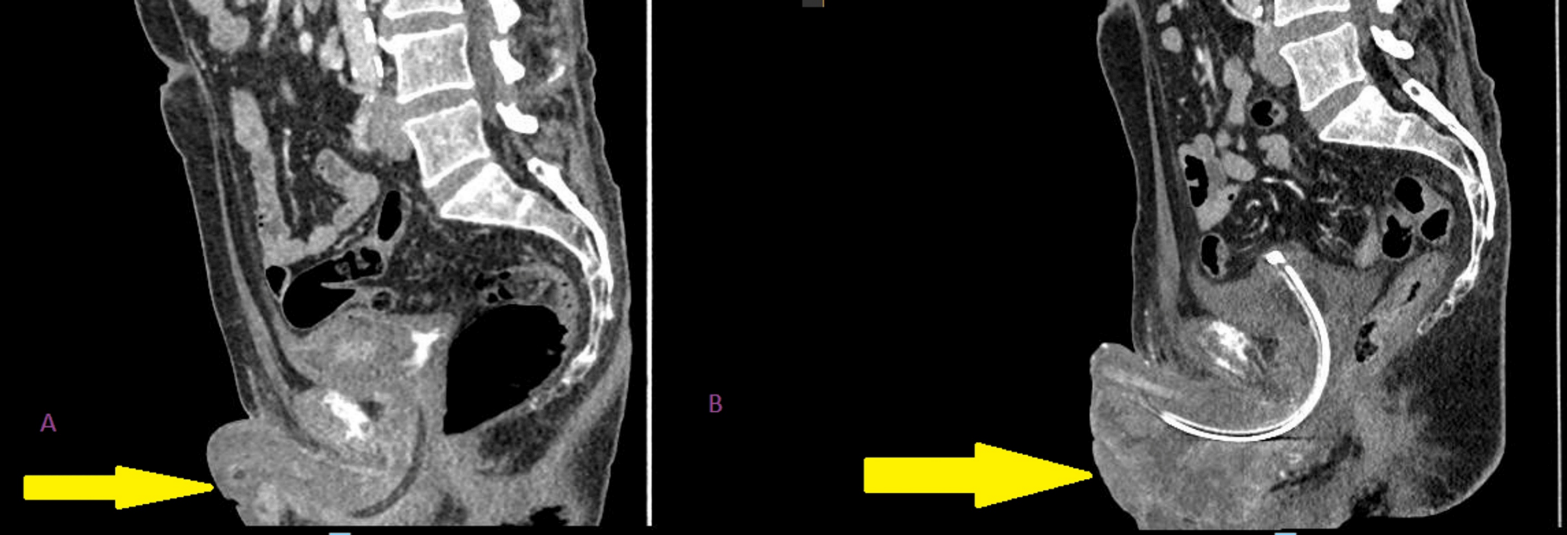
Cross-sectional sagittal image before radiotherapy in figure A and after radiotherapy in figure B showing the anatomy of the penoscrotal region post radiotherapy distorted by the huge mass with a urethral catheter in situ.

Magnetic resonance imaging (MRI) of his pelvis was then performed as shown in Figure 8, which showed a 73 mm poorly enhancing encapsulated mass within the inferior margin of the corpus spongiosum, not favoured to involve the urethra but involving the tunica albuginea, with a tiny amount of surrounding free fluid. The urethra was not clearly visualised due to compression from the mass. Re-staging CT scan done around the same time, as clearly demonstrated in Figures 9, 10, 11, had a similar conclusion regarding the penile mass along with enlarged iliac and retroperitoneal lymph nodes.

Bedside flexible cystoscopy was performed during the same admission which showed an irregular solid growth about 1.5 cm in length in the penile urethra occupying most of the lumen, bled easily on touch, and poor views of the bladder.

On imaging, it was postulated that the mass could possibly be another primary tumour such as a penile squamous cell carcinoma or sarcoma. However, the histological results and the view on urethrocystoscopy together made it most likely that this gentleman had a urethral recurrence of his urothelial carcinoma which extended into the penoscrotal area and eventually eroded through the skin. Differential diagnosis would be that this was from a metastatic deposit or extension of the main tumour. This then led to multiple fistulous tracts between the urethra and the skin. Repeat TURBT and resection of the penoscrotal mass were planned, however, whilst awaiting this, he was again admitted with the mass now fungating through the penoscrotal skin. A bedside biopsy of the scrotal mass was performed to facilitate quick histological diagnosis. This confirmed poorly differentiated urothelial carcinoma.

Systemically, the patient had declined at this point and did not wish for any further active treatment. He was then referred to palliative care and sadly passed away four weeks later.

## Discussion

It is rare for bladder cancer to metastasise to or directly invade through to the scrotum or penoscrotal subcutaneous tissue or skin. There are some reports of metastases to the testes. Patient survival after cutaneous metastases from transitional cell carcinoma is usually short and measured in months [9]. Invasion into the scrotum directly from urothelial carcinoma of either the bladder or urethra would be classed as locally advanced T4 disease and carry a poor prognosis.

One case report in 1998 showed solitary cutaneous metastasis of bladder cancer to the scrotum 18 months after TURBT [9]. It was treated with wide local excision and a course of chemotherapy, and the patient was disease-free 15 months after the second operation.

A case reported in 2015 by Norberg et al described a case of scrotal metastases of urothelial carcinoma which was treated with platinum-based chemotherapy with a durable complete response lasting 14 months after [10].

Another case reported in 2020 by Elumalai et al described a case of cutaneous metastasis of the bladder to the anterior pelvic wall, walls of the scrotum, and base of the penis diagnosed 8 years after the initial diagnosis of bladder cancer which was confirmed on histopathology [11].

There are multiple published cases of vesicocutaneous fistula occurring following treatment with radiation for pelvic malignancies including bladder [12,13]. A literature search did not yield any documented cases of vesicocutaneous fistula and scrotal involvement from direct extension of bladder cancer. The usual treatment would be urinary diversion to allow the fistulous tract to dry up prior to more extensive surgery if fit.

Muscle-invasive bladder cancer is known to be aggressive and in this case, some added high-risk features which may provide an explanation for early failure include squamous cell differentiation and positive lymph node. Another possible explanation for this local treatment failure in the index case is accelerated repopulation. The phenomenon is described as the rapid proliferation of cells during fractionated radiotherapy thereby leading to regeneration of the tumour and is believed to play a significant role in treatment failure [14,15]. This is usually as a result of prolonged treatment time as demonstrated in a study by Withers et al [16]. This is mitigated by accelerated fractionation schedules which aim to reduce the overall treatment time [14] or by radiation dose increment to compensate for tumor repopulation [16] but with the added risk of damage to normal tissue. 

## Conclusions

Early suspicion and organising cross-sectional imaging of non-resolving or atypical abscesses should be considered especially in the context of malignancy as we know muscle-invasive bladder cancer to be aggressive with poor prognosis despite treatment. Even with trimodal treatment (surgery, radiotherapy, and chemotherapy) treatment failure occurs. This is important in shared decision-making with patients so that they are well informed and can be referred to palliative care or other support teams in good time if failure occurs early.

Clinicians should remain vigilant in follow-ups, particularly in patients with urothelial carcinoma with persistent skin symptoms, and this should prompt early investigations to rule out fistulation and intervention carried out as appropriate.
